# Draft Whole-Genome Sequence of *Sphingobium* sp. 22B, a Polycyclic Aromatic Hydrocarbon–Degrading Bacterium from Semiarid Patagonia, Argentina

**DOI:** 10.1128/genomeA.00488-16

**Published:** 2016-06-02

**Authors:** L. Madueño, M. Macchi, I. S. Morelli, B. M. Coppotelli

**Affiliations:** a Centro de Investigación y Desarrollo en Fermentaciones Industriales, CINDEFI, (UNLP, CCT-La Plata, CONICET), La Plata, Buenos Aires, Argentina; b Comisión de Investigaciones Científicas de la Provincia de Buenos Aires (CIC-PBA), Buenos Aires, Argentina

## Abstract

*Sphingobium* sp. 22B is a polycyclic aromatic hydrocarbon–degrading strain isolated from Patagonia, Argentina, with capabilities to withstand the environmental factors of that semiarid region. The draft genome shows the presence of genes related with responses to carbon starvation and drying environmental conditions.

## GENOME ANNOUNCEMENT

*Sphingobium* sp. 22B is a Gram-negative, rod-shaped, chemoheterotrophic, strictly aerobic bacterium with a yellow colony color. It was isolated from soil contaminated with chronic polycyclic aromatic hydrocarbons (PAHs) from Pico Truncado, Argentina (1), and selected due to its great capacity of degrading PAHs as unique sources of carbon energy in mineral medium (LMM) and in phenanthrene microcosms assays (2). *Sphingobium* sp. 22B possesses resistance to soil environmental factors in semiarid Patagonia, including resistance to carbon starvation and drying conditions (1), and is useful in the autochthonous bioaugmentation process in Patagonian soil contaminated with PAHs.

*Sphingobium* sp. 22B was grown in R3 broth (3) at 24°C for 24 h, and high-quality DNA was extracted as described by Streit et al. (4) and Entcheva et al. (5). The genome sequence was obtained at INDEAR (Rosario, Argentina) following a whole-genome shotgun strategy on an Illumina HiSeq 1500 instrument using 2 × 100-bp reads and resulting in 400-fold genome coverage.

Reads were quality filtered with the Nextera XT Illumina protocol. *De novo* assembly of these reads was carried out with Illumina’s A5-miseq Assembly Pipeline version 2.0 platform (6). The RAST server version 2.0 (7) and the NCBI Prokaryotic Genomes Automatic Annotation Pipeline (PGAAP) (8) were used to predict and annotate the genes on the draft genome. Additionally, functional assignment of genes was performed by searching the KEGG and MetaCyc databases. The coding sequences (CDSs) were predicted by using a BLASTx alignment with the NCBI database (http://blast.ncbi.nlm.nih.gov).

The final assembly of *Sphingobium* sp. 22B was 5,367,847 bp in length, and the draft genome generated 107 contigs ranging from 220 bp to 665,822 bp, with a mean GC content of 61%. Based on RAST and PGAAP, a total of 5,021 CDSs, 5,006 genes, 4,829 proteins, 49 tRNAs, 4 rRNAs, and 121 pseudogenes were predicted.

An *in silico* search of stress genes showed that *Sphingobium* sp. 22B has 95 putative genes, with 133 CDSs related to polyhydroxybutyrate (PHB), glycogen, trehalose, glycine betaine synthesis and degradation, and exopolysaccharide (EPS) metabolism. The *Sphingobium* sp. 22B genome shows groups of two to five stress genes related to the same biochemical pathway that are involved in glycogen, trehalose, EPS, and glycine betaine metabolism. PHB genes are present in many copies both clustered and distributed throughout the genome.

Moreover, several genes for a complete set of enzymes involved in the degradation of aromatic compounds—29 CDSs of the PAH upper pathway and 55 CDSs of the PAH lower pathway—were recognized in the *Sphingobium* sp. 22B genome. Crucial genes for PAH degradation, such as those that codify for dioxygenase enzymes, were found: the alpha and beta subunits of aromatic-ring-hydroxylating dioxygenase, 2,3-dihydroxybiphenyl 1,2-dioxygenase, catechol 2,3-dioxygenase, and catechol 1,2-dioxygenase.

In summary, strain 22B demonstrates the presence of several PAH-degrading genes and genes related to desiccation response in Patagonia, and therefore the knowledge of gene dotation might be used to improve the bioremediation technologies in PAH-contaminated soils of semiarid Patagonia.

### Nucleotide sequence accession numbers.

This whole-genome shotgun project has been deposited in DDBJ/ENA/GenBank under the accession number LTAB00000000. The version described in this paper is the first version, LTAB01000000.
